# Exploring therapeutic responsiveness: a comparative textual analysis across different models

**DOI:** 10.3389/fpsyg.2024.1412220

**Published:** 2024-10-31

**Authors:** Dario Davì, Claudia Prestano, Nicoletta Vegni

**Affiliations:** Faculty of Psychology, Niccolò Cusano University, Rome, Italy

**Keywords:** responsiveness, psychotherapy research, gestalt therapy, psychodynamic therapy, systemic therapy

## Abstract

The purpose of this study is to examine therapeutic responsiveness across three different therapeutic models. The construct of responsiveness consists of two conceptual features: optimal responsiveness, which involves adapting therapist behavior to the unique therapeutic relationship, and appropriate responsiveness, a more refined concept. While aligned with interpersonal principles, the responsiveness construct challenges prevailing statistical methods by emphasizing the therapist’s adaptive responses. A comparative analysis of Gestalt, psychodynamic, and systemic therapies revealed unique patterns of responsiveness within each model, ranging from an emphasis on empathy and intuition to the significance of countertransference. Methodologically, a literature review and textual analysis using Atlas.ti allowed for nuanced exploration. The results also revealed a core commonality—"experience”—across these models, positioning responsiveness as an “extra-specific” factor amidst shared conceptual ground. In conclusion, this study sheds light on the nuances of responsiveness, which is central to advancing psychotherapeutic practice in an evolving landscape. An in-depth examination of the construct of responsiveness helps identify therapist characteristics that can be enhanced, enriched, and supported during training and supervision.

## 1 Introduction

Contemporary psychotherapeutic approaches emphasize the importance of emotional and relational competence in a universal way, including warmth, respect, empathy, acceptance, genuineness, reassurance, therapeutic alliance and hope. Responsiveness is generally a characteristic of caring relationships in which an affective bond with the caregiver is structured (Bowlby, 1982). In the therapeutic field, Rogers (1983) identified genuineness, acceptance and empathy as fundamental characteristics of a good therapist. In this scenario, responsiveness emerges as another essential characteristic of the therapist’s repertoire. The term ‘optimal responsiveness’ was introduced by Bacal (1985), suggesting that a responsive therapist adapts all his or her behaviors to the uniqueness of the therapeutic relationship, transcending both theory and technique. Stiles et al. (1998) and Stiles (2013) proposed the idea of ‘appropriate responsiveness’ to define the therapist’s ability to adapt responses to the client’s current state in order to achieve optimal benefit. This involves striving to always do the ‘right thing’ at the ‘right time’, while pursuing therapeutic goals and taking into account all the constraints, context and specific history of the relationship (Hayes et al., 1998). Responsiveness in psychotherapy can be seen as an ever-present feature of the therapist-patient relationship (Kramer and Stiles, 2015). Several studies (Baldwin et al., 2007; Zuroff et al., 2010) show that therapist responsiveness can be an important contributor to treatment effectiveness. Broadly speaking, therapist responsiveness is inherent to human interaction and is rooted in a positive attitude towards the patient and the ability to personalize the therapeutic experience through an empathic lens. It involves acknowledging emotions, providing information, validating client successes, promoting self-care and social support, and building alliances (Li et al., 2020). The ability to be responsive is crucial in matching client needs with the most appropriate procedures and techniques.

Responsiveness is a similar construct to attachment, therapeutic alliance and countertransference. Research by Wallin (2007) on attachment in psychotherapy also supports the role of responsiveness in enhancing the therapeutic bond. He emphasizes that the therapist’s ability to respond adaptively to the patient’s attachment patterns fosters a secure base within the therapeutic relationship. Responsiveness to the patient’s attachment needs, particularly in cases where early attachment experiences were disrupted or insecure, plays a key role in facilitating psychological healing and growth (Mallinckrodt and Jeong, 2015). According to Wiseman and Egozi (2021), attachment theory is related to the construct of responsiveness in that it provides a good empirical base, encourages a developmental reading of the construct, and makes responsiveness a common factor across different theoretical orientations.

Recent studies emphasize the role of responsiveness in managing countertransference, which has been identified as a critical element in psychodynamic practice (Gelso, 2021). Responsiveness helps therapists navigate their emotional reactions to patients, fostering a therapeutic alliance that supports the patient’s exploration of unconscious material (Colli et al., 2014). Safran and Muran (2020) suggest that responsiveness not only helps in managing ruptures in the therapeutic alliance but also plays a pivotal role in repairing these ruptures, contributing to the overall efficacy of treatment. This adaptive process of rupture and repair underscores the need for therapists to remain attuned to the evolving dynamics of the therapeutic relationship. Responsiveness in these moments requires therapists to display emotional flexibility and to hold both the therapist’s and patient’s emotions in mind (Safran et al., 2014).

Moreover, Holmes (2014) emphasizes the connection between responsiveness and reflective functioning, where the therapist’s ability to understand and interpret the patient’s internal mental states contributes to a more nuanced and flexible therapeutic stance. Reflective functioning allows therapists to respond sensitively to the emotional content of the therapeutic relationship, promoting the patient’s capacity for self-reflection and mentalization (Fonagy and Allison, 2018). In this context, responsiveness goes beyond empathy and involves the ability to maintain a reflective space where the patient can explore previously unconscious aspects of their internal world (Allen et al., 2008). Recent contributions by Cortina and Liotti (2021) extend the notion of responsiveness into a neurobiological context, where they argue that therapist attunement activates the patient’s social engagement system. This responsiveness has a calming effect on the patient’s nervous system, helping to regulate affective states and making it easier for patients to engage in deeper therapeutic work. Neurobiological research in psychodynamic therapy has increasingly pointed to the role of co-regulation, where the therapist’s responsiveness serves not only an emotional but also a physiological regulatory function (Schore, 2019).

While responsiveness is consistent with general principles of interpersonal relationships, it differs from the conventional sense of common factors in psychotherapy (Davis and Hsieh, 2019; Norcross and Wampold, 2011). On the one hand, it poses a challenge to researchers attempting to model clinical practice with linear, ballistic logic and the statistical methods prevalent in psychotherapy research (Stiles, 2013), while on the other it may be more intuitive to clinicians. Responsiveness challenges the assumption that some treatments can be universal for all clients, while emphasizing the therapist’s adaptive responses to specific client characteristics and different clinical scenarios.

## 2 Comparing different clinical models

The role of responsiveness across various therapeutic approaches was elucidated by the American Psychological Association (Watson and Wiseman, 2021), offering a comprehensive overview of effective interventions in psychotherapy. In alignment with the most recent APA publication, this study endeavors to scrutinize the evidence for convergences and divergences among three distinct clinical models: Gestalt Therapy, Systemic and Psychodynamic Therapy. The concept of responsiveness is prominently featured in relational, humanistic, and psychodynamic paradigms. Conversely, cognitive-behavioral approaches employ the construct of *adaptation* (Norcross and Wampold, 2019). From a broader standpoint, the assessment of responsiveness was recently undertaken using the *Patient’s Experience of Attunement and Responsiveness Scale* (PEAR, Snyder and Silberschatz, 2017; Zittel Conklin and Westen, 2003). The three different approaches are described below.

### 2.1 Gestalt therapy

Gestalt therapy is a humanistic approach rooted in phenomenology, underscores the significance of the therapist’s empathy and intuition. It delves into phenomenological exploration, focusing on the experiential processes arising within a relational field. The Gestalt therapist tracks the client’s emerging experience through the concept of “being-with” (Frank, 2020; Spagnuolo Lobb, 2019; Snyder and Silberschatz, 2017), co-creating the contact boundary between therapist and client in the session’s present moment (Snyder and Silberschatz, 2017). The Aesthetic Relational Knowledge Scale (ARKS, Spagnuolo Lobb et al., 2024, 2023a, 2023b, 2022) further contributes with theoretical and clinical insights into exploring responsiveness and emphasizing therapist intuition, resonance, and embodied empathy.

### 2.2 Systemic therapy

Responsiveness is understood as a dynamic process that not only attends to individual family members but also addresses the relational patterns and interactions within the family system. Recent research emphasizes the importance of the therapist’s attunement to systemic dynamics, particularly in recognizing and responding to family hierarchies, roles, and communication patterns (Lebow, 2022; Nichols and Davis, 2020; Sandberg et al., 2016). According to Lebow (2022), systemic therapy requires a high degree of flexibility and responsiveness to the changing relational dynamics, with interventions that address both individual and collective needs. This involves working with the family as a unit and understanding how emotional, cognitive, and behavioral patterns are interconnected across generations (Karam et al., 2022). The therapist’s responsiveness in systemic therapy also includes the ability to manage emotional intensity, regulate interactions, and facilitate the family’s ability to co-regulate. Carr (2009) emphasizes the importance of therapists adopting a neutral but engaged stance, allowing the family system to reorganize and adapt through processes like circular questioning, reframing, and relational hypotheses. In more recent approaches, systemic therapists integrate aspects of mindfulness and emotional regulation, as seen in Emotionally Focused Family Therapy (EFFT), where the therapist attunes to both individual emotional experiences and family dynamics, fostering secure attachment and systemic change (Johnson and Sanderfer, 2016; Furrow et al., 2022). Additionally, the use of technology in systemic therapy has expanded therapist responsiveness in new ways. Teletherapy, increasingly utilized during the COVID-19 pandemic, has been shown to enable effective systemic interventions, where therapists maintain responsiveness to family dynamics despite physical distance (Aviram and Nadan, 2022; McLean et al., 2021). This evolution underscores the adaptability of the systemic approach in diverse contexts, further supported by tools like the Genogram and the Systemic Family Assessment (SFA), which enhance the therapist’s capacity to understand and respond to the complex interplay of familial relationships (Hardy and Laszloffy, 1995).

### 2.3 Psychodynamic therapy

Tishby (2021) highlights how the construct of responsiveness is intricately linked to other key features of the therapist-patient relationship, such as mutual recognition (Benjamin, 1990a) and implicit relational knowledge (Stern, 1998). Mutual recognition refers to the therapist’s capacity for empathic attunement to the needs of the patient, fostering a therapeutic space where both the therapist and patient are acknowledged as distinct yet interconnected individuals. This concept aligns closely with the relational turn in psychoanalysis, emphasizing the importance of the therapist’s authentic and adaptive responses in co-creating the therapeutic relationship (Mitchell, 2000).

Implicit relational knowledge, as described by Stern (1998), involves the therapist’s ability to engage in non-verbal, procedural forms of knowing, often unconsciously, which guide the therapeutic process. This type of responsiveness includes subtle, embodied interactions that occur beneath the level of conscious awareness, facilitating deeper emotional attunement and engagement. Implicit relational knowing is considered crucial for creating a corrective emotional experience within psychodynamic therapy (Lyons-Ruth, 2019).

## 3 Methods

To conduct a comparative analysis of different treatment models, a comprehensive literature search was performed across multiple databases. Initially, grey literature was consulted through search engines such as Google Scholar and ScienceDirect. Following this, a search for peer-reviewed scientific articles was conducted in PubMed and PsycINFO, yielding a total of 6,736 articles. From this pool, *N* = 197 articles and book chapters were selected. The first phase of the search focused on articles and book chapters addressing the general construct of responsiveness in psychotherapy, with all sources not directly addressing this specific topic excluded. The keywords “responsiveness” OR “responsive psychotherapist” AND “psychotherapy” were employed, resulting in *N* = 57 articles selected from a total of *N* = 2,101. In the second phase, additional keywords, including “responsiveness” OR “responsive psychotherapist” AND “Gestalt therapy,” were applied, yielding 50 articles from a total of *N* = 1,410. Subsequently, the keywords “responsiveness” OR “responsive psychotherapist” AND “systemic therapy” were used, leading to the selection of *N* = 45 articles from a total of *N* = 1,492. Finally, the keywords “responsiveness” OR “responsive psychotherapist” AND “psychodynamic therapy” were applied, resulting in the selection of *N* = 45 articles from a total of *N* = 1,733. Data analysis was conducted using Atlas.ti, a qualitative data analysis tool based on Grounded Theory.

## 4 Results

Researchers, using the textual analysis software Atlas.ti, highlighted themes that emerged from the articles and categorized them into broader labels and subcategories. Therefore, textual analysis revealed *N* = 6 families (1. Here and Now, 2. Intersubjectivity, 3. Client’s centered, 4. Empathic therapist, 5. Experience, 6. Positive Attitude) and *N* = 13 categories (a. Emerging, b. Moment by moment, c. Human Interaction, d. Mutual Regulation, e. Personalization, f. Client’s need, g. Client Experience, h. Therapist experience, i. Both Experience, j. Positive Response, k. Positive communication). All categories were then analyzed according to four different perspectives: general (G), Gestalt Therapy (GT), systemic therapy (ST), and psychodynamic therapy (PT). Results are shown below (Table 1).

**Table 1:** The therapeutic responsiveness.

1. Here and Now *Responsiveness emerges in the ‘hic et nunc’ of interaction. It consists of a spatial and a temporal dimension*	
*a. Emerging* *(Responsiveness as an emergent event)* *G: “Therapist responsiveness is defined as therapist behavior being influenced by emerging context”* (Kramer and Stiles 2015, p. 277). *GT:“The intuitive experience of the therapist that emerges from the phenomenological field created in a meeting between therapist and client”* (Spagnuolo Lobb et al., 2022, p. 10) *ST: “Some positive finding are emerging”* (Jenkins and Asen, 1992, p. 83) *PT: “These competencies emerging in a psychotherapist-in-training, facilitated by an intense interaction with a supervisor”* (Sarnat, 2010, p. 20)	*b. Moment by moment* *(Responsiveness is built moment by moment)* *G: “I liken the moment-to-moment decisions I make about listening and speaking when I’m with my patient”* (Geller, 2011, p. 9) *GT: “Therapist becomes present for their client”* (Denham-Vaughan, 2005, p. 4) *ST: “Monitoring the patients’ moment-to-moment verbal communications with the therapist”* (Miller-Bottome et al., 2018, p. 5). *PT: “The ways in which it impacts the moment-to-moment interaction with patients”* (Talia et al., 2020, p. 2)

2. Intersubjectivity *The relevant relational context*	
*a. Human interaction* *(Responsiveness arises from an interaction between human beings)* *G: “Psychotherapy is a special form of interpersonal interaction”* (Hatcher, 2015, p.748). *GT: “Envisages that the Self exists in interaction with others”* (Raffagnino, 2019, p. 70) *ST: “An important aspect of family therapy concerns the ways in which family members relate to the therapist”* (PettyJohn et al., 2020). *PT: “Psychodynamic psychotherapy, like all psychotherapeutic approaches, requires interpersonal skills, such as building a relationship with the client”* (Sarnat, 2010).	*b. Mutual regulation* *(Responsive interaction is mutual)* *G: “Both the dynamic dyadic process and the mutual influence and feedback loop suggested in the concept of responsiveness”* (Li et al., 2020). *GT: “The meeting between therapist and client”* *ST:* “*In the middle and especially the final stage of therapy most therapy sessions were characterized by a sense of warmth and mutual collaboration”* (Cirasola et al., 2022, p. 13). *PT: “The analytical relationship as a particular intersubjective context (or two-way system) of mutual influence between analyst and patient”* (Favaretto et al., 2022, p.48)

3. Client’s centered *Responsiveness is a client-centered characteristic*	
*a. Personalization* *(Treatment customization)* *G: “What treatment, by whom, is most effective for this individual with that specific problem and under what circumstances?”* (Paul, 1967, p. 111) *GT: “Singularity”* (Day, 2016) *ST: “Person-centered democratic practice, as opposed to clinician-led paternalistic approaches”* (Falicov et al., 2021, p. 671) *PT: “Countertransference patterns are systematically related to patients’ personality pathology across therapeutic approaches”* (Betan et al., 2005, p. 890).	*b. Client’s need* *(Centering on patient needs)* G: “*We include the construct of “responsiveness” in our scale because we wish to emphasize the importance of the therapist’s responsiveness to a patient’s therapeutic needs*” (Snyder and Silberschatz, 2017). GT: *“For some clients this was the only possible way of receiving such a service.”* (Edirippulige et al., 2013, p. 378) ST:*“Therapists should attend to the client’s difficulties”* (Zahl-Olsen et al., 2020, p.1) PT: *“Focused on client perspectives”* (Jones et al., 2020, p. 2).

4. Empathic therapist *The responsive therapist shows empathy*	G: *“Therapists rating their own empathy (empathic resonance)”* (Elliott et al., 2011, p.44). GT: “*The capacity to feel at a bodily level what the other feels”* (Spagnuolo Lobb et al., 2022, p.3) ST: *“The therapist showed empathy and validation of the patient’s difficulty”* (Cirasola et al., 2022, p. 2) PT: *“To demonstrate that therapists were with them and empathizing”* (Anvari et al., 2020, p.908)

5. Experience *Experience contributes to the history of the members of the therapeutic dyad*		
*a. Client experience* *G: “One important difference between clients is their readiness to take a reflective stance on their problems and experiences” (*Penttinen et al., 2017, p. 1) *GT: “Understanding of the client’s internal experience”*(Imes et al., 2002. p.1369) *ST: “Asking clients about their own unique experiences”* (PettyJohn et al., 2020, p. 317). *PT: “Identifying recurrent patterns of action/feelings/experiences”* (Zeeck et al., 2019, p. 390)	*b. Therapist experience* *G: “The second source of variability in the alliance is related to the therapist”* (Baldwin et al., 2007, p. 843) *GT: Personal Therapy Experience and Training* (Steiner 13) *ST: “As to how therapists experience the transition to online therapy”* (Machluf et al., 2022 p.146). *PT: “The therapist’s experience during the session is particularly meaningful”* (Rober, 2021, p. 6)	*c. Both experience* *G: “Phenomenological flow of experience is a central focus for psychotherapeutic work”* (Silva and Sousa, 2022). *GT: “The therapist can (a) follow the emerging experience of the client*, (p. 220) *ST: “The present study aims to understand experience of therapy and processes”* (Collyer et al., 2021 p. 318) *PT: “Psychotherapy as a correcting experience”* (Rief, 2021, p. 117)

6. Positive Attitude *A positive approach to treatment*	
*a. Positive response* *(Therapists respond to clients in the best possible way)* *G: “The comprehensive assessment of responsiveness, called the positive therapeutic atmosphere”* (Kramer and Stiles, 2015) *GT: “The vision of a positive future”* (Bocian, 2020, p. 367). *ST: “Therapists’ positive attitudes toward online couples’ therapy”* (Machluf et al., 2022). *PT: “Although several studies have hypothesized that therapists may report positive feelings due to defensive processes or lack of awareness of their own negative feelings toward patients with personality disorders”* (Bhola and Mehrotra, 2021).	*b. Positive communication* *(Constructive and positive dialogue)* *G: “Positive regard and affirmation”* (Norcross, 2002). *GT: “Improved their level of empathic communication”* (Steinmair et al., 2020, p. 10) *ST: “Facilitating positive family emotional climate”* (Wood et al., 2021, p.11) *PT:“The secure frame offers the patient the safest and most open conditions for free and unencumbered communication”* (Troise and Quinn, 1991).

## 5 Discussion

As indicated above, examination of the selected articles reveals categories and subcategories that explore the construct of responsiveness in general and specific to the approach. In this section we describe and compare the models, highlighting similarities and differences.

### 5.1 Here and now

The category we have termed “*emerging*” indicates space, context (Kramer and Stiles, 2015) and the phenomenological field (Spagnuolo Lobb et al., 2022). This emergent responsivity of the therapist has a positive connotation (Jenkins and Asen, 1992) and is seen as intense and facilitating therapeutic work (Sarnat, 2010). Furthermore, the therapist’s responsiveness is constructed ‘*Moment by moment*’, within a dialogue. This label emphasizes the processual and temporal dimension of responsiveness.

### 5.2 Intersubjectivity

The literature review shows that three approaches, Gestalt, systemic and psychodynamic, share the theoretical and epistemological framework of the relational model (Mitchell, 2000; Benjamin, 1990b/1999). Thus, the characteristics that emerge emphasize that responsiveness develops in a human context, consisting of real people who are in constant negotiation with each other. From a general perspective, it is a particular form of ‘*human interaction*’ linked to a context that has care as its goal (Silva and Sousa, 2022; Hatcher, 2015). It is in this category that the human qualities of the therapist emerge, his interpersonal skills (Sarnat, 2010) which, in the case of systemic therapy, enable him to relate to the family system (PettyJohn et al., 2020). Another dimension explored is ‘*Mutual regulation’*, which describes the processuality of interaction (Favaretto et al., 2022; Li et al., 2020; Safran et al., 2001).

### 5.3 Client’s centered

This category has two sub-categories: ‘*Personalization*’ and “*Client’need*.” In terms of personalization, being responsive means reserving a specific treatment for a specific problem and in specific circumstances (Paul, 1967), which implies attention to the patient’s subjectivity (Day, 2016). In a psychodynamic perspective, this specificity takes the form of the analyst’s countertransference (Betan et al., 2005) and its framing in a personality theory. Adaptation according to the systemic approach, on the other hand, focuses on the development of the patient and his or her development as a person. From a general perspective, caring for the patient means giving importance to the patient’s needs, which therefore implies offering a specific service (Snyder and Silberschatz, 2017) by understanding the patient’s perspective (Zahl-Olsen et al., 2020) and his or her particular difficulties (Jones et al., 2020). Empathy is also a feature of the responsiveness construct.

### 5.4 Empathic therapist

This label implies that the therapist’s responsiveness is directly related to his or her empathy (Spagnuolo Lobb et al., 2022; Cirasola et al., 2022; Brøsholen et al., 2022; Anvari et al., 2020; Hatcher, 2015; Bourke and Grenyer, 2010).

### 5.5 Experience

This is an important dimension of the construct. The literature review shows that responsiveness consists of the “*Client’s experience*,” the “*Therapist’s experience*” and ‘*both experiences*’. The *‘client’s experience*’ is understood as the activation of the client’s internal (Imes et al., 2002) and unique dimension (PettyJohn et al., 2020). While the “*therapist’s experience”* seems to be very much related to training and supervision experiences (Steinmair et al, 2020) that enhance his ability to reason about his work (Machluf et al., 2022; Rober, 2021). *Both’s experience*’ is considered the main phenomenological approach to responsiveness (Silva and Sousa, 2022). From a Gestalt perspective, the responsive therapist follows the emergence of the patient’s experience step by step. Therapy is therefore seen as a transformative experience (Rief, 2021).

### 5.6 Positive attitude

This label is understood as a positive atmosphere (Kramer and Stiles, 2015) that the therapist helps to create. From the Gestalt perspective, responsiveness is connoted by a positive function of the future that promotes hope” (Bocian, 2020). From a systemic perspective, responsivity focuses on therapeutic work aimed at developing ‘a positive emotional family climate’ (Wood et al., 2021, p. 11), and from a psychodynamic perspective it coincides with a positive attitude in assessing personality structure and diagnosis (Bhola and Mehrotra, 2021) aimed at enhancing the patient’s psychic resources. Responsiveness is also associated with *“positive communication*” (Steinmar, 2020; Norcross, 2002; Troise and Quinn, 1991) that characterizes the caring relationship.

## 6 Comparing models and learning from experience

### 6.1 Comparing three different models

Analysis of the construct revealed similarities and differences between the three approaches, which share the fact that responsivity emerges in a relational context and in the ‘here and now’ of interaction. In Gestalt psychotherapy in particular, responsivity is linked as an emergent fact to the intuitive experience of the therapist, and the therapeutic context is constituted as a phenomenological field (Spagnuolo Lobb et al., 2022). Systemic and psychodynamic approaches are similar in that they see the setting as an intense and positive context in which patient-therapist communication plays a crucial role, but in the psychodynamic approach it is the countertransference that plays a key role (Coyne et al., 2019; Sarnat, 2010; Bein et al., 2000; Hayes et al., 1998; Jenkins and Asen, 1992). Indeed, moment-to-moment construction occurs in the presence of the therapist, according to the Gestalt approach (Denham-Vaughan, 2005, p. 4), with intense communication and interaction for systemic and psychodynamic models (Talia et al., 2020; Miller-Bottome et al., 2018). As mentioned above, the epistemological reference model for all three approaches is the relational model (PettyJohn et al., 2020; Raffagnino, 2019; Sarnat, 2010). And mutual regulation between patient and therapist as well as the relationship model is a common feature of Gestalt, systemic and psychodynamic approaches. A responsive attitude is primarily patient-centered and implies personalized treatment and attention to the client’s needs (Bacal and Herzog, 2003; Norcross and Wampold, 2019; Snyder and Silberschatz, 2017; Bacal and Herzog, 2003). According to the familial model, the personalization of the intervention takes the form of acknowledging the patient’s difficulties, excluding a paternalistic and authoritarian intervention, but a dialogical one (Falicov et al., 2021; Zahl-Olsen et al., 2020; Davis and Hsieh, 2019). According to the psychodynamic model, responsiveness makes the intervention specific by paying attention to the patient’s diagnostic assessment and personality structure (Jones et al., 2020). Thus, unlike psychodynamic and Gestalt approaches, which tailor the intervention solely to the characteristics of the patient, the family approach also takes into account the social context in which the client is embedded. The study of specific therapeutic models has highlighted the unique nuances of responsiveness within each approach, from the emphasis on empathy and intuition in Gestalt therapy, to the importance of countertransference in psychodynamic practice, to communication and dialogue in the systemic approach.

### 6.2 Experience as a common ground

Integrating the insights of different therapeutic approaches revealed a common element: experience. Created in the individual’s interaction with the environment, experience is understood and nurtured in the therapeutic ‘in-between’. It is classified as therapist experience, client experience and mutual experience. This common feature emphasizes responsiveness as the ability to adapt a therapeutic pathway to the specific context and characterizes the three approaches – Gestalt, Systemic and Psychodynamic. Despite this commonality, there is a paradox in the experiential dimension: an aspect common to the three approaches but subject to specific evaluations. Indeed, if the psychotherapeutic experience is the ‘common field’ within which responsiveness takes shape, it is at the same time a unique and unrepeatable experience because it is co-created by the specific patient-therapist dyad. This dimension therefore escapes any analysis that is not linked to the specific patient-therapist relationship under investigation. Responsiveness can therefore be considered as an extra-specific factor that stands out in the complexity of psychotherapeutic practice. In general, it is therefore important to recognize the differences between patients (Penttinen et al., 2017). Responsiveness as a common factor is manifested in the therapist’s ability to adapt the therapeutic process to the specific context.

## 7 Conclusion

The present study represents an in-depth study of the construct of responsiveness, which has recently been the subject of analysis by researchers (Calaboiça et al., 2024), in order to highlight which characteristics of the therapist can be effective in treating patients (Lauritzen et al., 2023). In our opinion, a detailed analysis of the construct is necessary in order to study a very broad concept consisting of several sub-areas that can be studied in detail and constitute new and more effective measurement tools (Esposito et al., 2024; Spagnuolo Lobb et al., 2024, 2023b; Tanzilli et al., 2024). Furthermore, a comparison of models is now more important than ever in order to identify individualized and integrated psychotherapeutic pathways aimed at increasing the effectiveness of interventions. The starting point is the psychotherapist’s experience and its characteristics; exploring the clinician’s way of working allows new insights to be gained through targeted supervision and training (Fuchs and Stemberger, 2022; McWilliams, 2022). In our opinion, new research should further explore the therapist’s experience in relation to the increasingly complex everyday life (Hill and Norcross, 2023; Cervellione et al., 2023). In conclusion, as the therapeutic landscape continues to evolve, understanding and researching responsiveness will remain fundamental to improving therapeutic outcomes and advancing the field of psychotherapy.

## 8 Limits

The study possesses certain limitations that warrant consideration. First, it predominantly focuses on three specific therapeutic models—Gestalt Therapy, Psychodynamic Psychotherapy, and Systemic Therapy. While these models provide valuable insights, the study’s findings may not be universally applicable to a broader spectrum of therapeutic methods and variations within the chosen models. Second, there is a potential bias introduced by the study’s reliance on specific keywords and the concentration on English-language literature. This approach might result in overlooking relevant studies that employ different terminology and may not adequately encompass valuable contributions available in languages other than English. Third, despite the study’s incorporation of both qualitative and quantitative analyses, its reliance on established theoretical frameworks and tools like Atlas.ti could simplify the nuanced and subjective nature of responsiveness. This approach might not fully capture the complexity of therapist behaviors and could potentially overlook diverse perspectives emerging from newer theoretical frameworks.

## Data Availability

The data supporting the conclusions of this article will be made available by the authors, upon reasonable request. Requests to access these datasets should be directed to DD, dario.davi@unicusano.it.
